# Serum miR-146a and miR-155 as Predictors of Virological Control After Entecavir Withdrawal in HBeAg-Positive Chronic Hepatitis B Patients

**DOI:** 10.5152/tjg.2025.24685

**Published:** 2025-03-18

**Authors:** Ming Li Xu, Li Huan Wang

**Affiliations:** 1 Department of Infectious Diseases, Jiashan County First People’s Hospital, Zhejiang, China

**Keywords:** Chronic hepatitis B, entecavir, HBeAg, miR-146a, miR-155

## Abstract

**Background/Aims:**

Distinct virological relapse (VR) has occurred after stopping the use of nucleos(t)ide analogues treatment in patients with chronic hepatitis B (CHB) who are positive for Hepatitis B e antigen (HBeAg). Here, we aimed to identify serum miR-146a and miR-155 in HBeAg-positive patients with CHB and their relationship with VR after entecavir (ETV) discontinuance.

**Materials and Methods:**

Ninety-eight patients with CHB and positive for HBeAg before initiating treatment were analyzed. Virological relapse was defined as 2 consecutive examinations (3 months apart) after treatment discontinuance, both showing an HBV DNA load of more than >2000 IU/mL.

**Results:**

Compared to control, CHB patients were observed with increases in serum miR-146a and miR-155 before starting ETV treatment (*P* < .001). There were 52 patients (53.1%) experiencing VR at 12 months after discontinuance of ETV treatment. The VR group was demonstrated with increases in serum miR-146a and miR-155 than the non-VR group before starting ETV treatment (*P* < .001). Whether at the beginning of ETV discontinuance or at 12 months after ETV discontinuance, the VR group was noted with elevations in serum miR-146a and miR-155 than the non-VR group (*P* < .001).

**Conclusion:**

The findings demonstrated that miR-146a and miR-155 levels in the circulation may be promising assessment tools for identifying sustained-virological responders after ETV discontinuance in CHB patients.

**Main Points**

- The cumulative virological relapse rate of hepatitis B e antigen-positive patients with chronic hepatitis B (CHB) infection was 53.1% (52/98) at 12 months after discontinuance of entecavir (ETV) treatment.
- Serum miR-146a and miR-155 levels were higher in patients with relapse of HB infection than in those with sustained virological response before initiating ETV treatment, after 12-month consolidation therapy, and after 12-month ETV discontinuance.
- Circulating miR-146a and miR-155 may be promising assessment tools for identifying sustained-virological responders after ETV discontinuance in patients with CHB infection.

## Introduction

Hepatitis B virus (HBV) infection is a major public health threat that chronically affects about 296 million people and causes an estimated 820 000 deaths worldwide in 2019.^1^ Although the World Health Organization set a goal of eliminating HBV as a public health problem by 2030, the annual mortality of HBV is projected to increase by 39% from 2015 to 2030 if no significant advances are made to develop a finite HBV treatment.^2^ Chronic hepatitis B (CHB) has 2 different serologic types, hepatitis B e antigen (HBeAg)-positive form and HBeAg-negative form. Hepatitis B e antigen is a vital hallmark for active replication of HBV, and HBeAg seroconversion is considered to be the endpoint of oral antiviral therapy for HBeAg-positive CHB.^3^

Antiviral therapy for HBV with oral nucleos(t)ide analogs (NAs), such as entecavir (ETV) and tenofovir, is the current first-line therapy with improved clinical outcomes for patients with CHB. Some HBeAg-positive patients achieving stable HBeAg seroconversion can be safely withdrawn from treatment.^4^ A “functional” cure, defined as sustained loss of HB surface antigen (HBsAg), is a critical clinical milestone in antiviral therapy for CHB but is rarely achieved.^5^ Available data show that only 10% to 20% of patients can achieve sustained loss of HBsAg after stopping NA treatment, with 50% to 80% of patients experiencing virological relapse (VR) and 40% to 55% of patients requiring retreatment with NAs.^6^ Current treatment guidelines suggest that NA therapy could be discontinued following 6 to 12 months consolidation therapy if patients have sustained HBeAg seroconversion in HBeAg patients.^3^^,^^7^ However, there is no consensus about the duration of consolidation therapy with seroconversion of HBeAg during antiviral treatment for CHB. Predictors of sustained virological response have been studied to optimize treatment endpoints for minimizing the risk of VR after NA therapy, involving serum HBsAg load and HB core-related antigen level.^8^

In comparison to other known predictors, microRNAs (miRNAs), a group of known small non-coding RNAs that can be stably expressed in body fluids, are not only identified as important predictors of virological response but also linked to hepatocellular carcinoma risk following NA treatment.^9^^,^^10^ miR-146a is a widely studied regulator in HBV replication, and its upregulation caused by the HBV X protein has been found to promote CHB progression.^11^ miR-155 has been identified to be positively correlated with the immune activation of CD4+ and CD8+ T-cells, thus inducing immune activation and disease progression in CHB patients.^12^ An Egyptian population study demonstrated that miR-146a A/G (rs57095329) and miR-155 T/A (rs767649) single nucleotide polymorphisms may increase the risk of developing CHB.^13^ However, whether NA treatment can be safely discontinued and biochemical factors related to virological response and reflecting the optimal duration of consolidation therapy after ETV discontinuance are not well defined. In this study, we investigated the expression patterns of miR-146a and miR-155 in HBeAg-positive patients with CHB and explored the role of serum miR-146a and miR-155 in sustained virological response after ETV discontinuation in HBeAg-positive CHB.

## Materials and Methods

### Participants

The study recruited patients who started treatment with ETV for CHB from January 2018 to June 2020, and who were positive for HBeAg before initiating treatment. Inclusion criteria: i) HBsAg (HBsAg ≥ 0.05 IU/mL) positive for >6 months; ii) HBeAg exceeding 1 S/CO prior to antiviral treatment; iii) oral administration of a tablet of ETV (0.5 mg per day) and completion of 12-month follow-up after ETV discontinuance; iv) meeting the criteria for drug discontinuance recommended by the Asian Pacific Association for the Study of the Liver (APASL) (2015)^14^: patients discontinued ETV when reaching both HBV DNA negative conversion and HBeAg seroconversion for at least 12 months; and v) >18-year-old patients. Exclusion criteria: i) imaging evidence of liver cirrhosis, and/or liver biopsy (Ishak fibrosis scores >4) before initiating treatment; ii) coexistence of viral hepatitis A, C, D, or E, autoimmune hepatitis, or cholestatic liver disease; iii) patients with a history of resistance to any kind of NA; iv) those with a history of off-treatment VR; v) use of immunomodulators in the past 6 months; and vi) pregnant women. The study protocol complied with the principles of the Declaration of Helsinki and was approved by the Ethics Committee of Jiashan County First People’s Hospital (approval number: 2024112, date: December 26, 2024). Written informed consent was obtained from the patient(s) who agreed to take part in the study.

### Blood Sample Collection at Indicated Time Points

Fasting blood samples were obtained in the morning after overnight fasting from healthy volunteers at their physical examinations. These samples included patients before starting ETV treatment (the baseline) and off-treatment time points, including at 0, 3, 6, 9 and 12 months after ETV cessation. The blood samples were processed by centrifugation (2000 × g, 10 minutes) to obtain the serum.

### Biochemical Examinations

The alanine aminotransferase (ALT) levels were determined by the Siemens ADVIA 2400 automatic biochemical analyzer (Siemens Healthcare Diagnostics, Deerfield, IL, USA). The upper limit of normal value of ALT was 40 U/L. The HBsAg and HBeAg levels were quantified by chemiluminescent microparticle immunoassay (Architect i2000 analyzer; Abbott Laboratories, Illinois, USA). The HBV DNA load was determined by using the LightCycler® 480 Real-Time PCR System (Roche Diagnostics, Basel, Switzerland).

### MicroRNA Expression Measurements

The total RNA extracted from patient serum was added with 200 amol of artificial Caenorhabditis elegans miR-39 (cel-miR-39, Sigma-Aldrich, USA) as a spike-in control. Reverse transcription polymerase chain reaction and quantitative polymerase chain reaction were applied to examine miRNA expression by using the TaqMan® MicroRNA Reverse Transcription Kit and TaqMan® miRNA Assays (Invitrogen) on the LightCycler® 480 Real-Time PCR System (Roche). Expression of hsa-miR-16-5p was also examined as a reference miRNA. The used forward primer sequences for miR-146a, miR-155, cel-miR-39, and miR-16-5p are: 5’-TGAGAACTGAATTCCATGGGTT-3’, 5’-GCCGAGTTAATGCTAATTGTG-3’, 5’-GTGTCACCGGGTGTAAATC-3’, and 5’-CGCGTAGCAGCACGTAAATA-3’. The universal reverse primer sequence is 5’-TGGTGTCGTGGAGTCG-3’. The results were calculated by comparative Ct with the formula 2^-ΔΔCt^.

### Definitions

Sustained off-treatment virological response, also termed non-VR (non-VR) in this study, was deemed as undetectable serum HBV DNA for at least 12 months after stopping oral ETV treatment. Virological relapse was defined as 2 consecutive examinations (3 months apart) both showing serum HBV DNA >2000 IU/ml.^14^

### Statistical Analysis

Quantitative variables were summarized as a mean with standard deviation (SD). Differences between HBeAg-positive CHB patients and healthy controls, non-VR and VR patients were assessed by using dependent or independent sample *t*-tests. Count data are presented as total number with percentages (%) and analyzed using the chi-squared tests. A prediction model using receiver operating characteristic (ROC) curves and its summary statistics [area under the curve (AUC)] was analyzed in the target population (VR group) differing from the source population (non-VR group). GraphPad Prism software was employed to examine group differences and time-dependent trends version 8.0 (GraphPad Software, San Diego, CA, USA). All reported *P* values are two-sided, with a cutoff value of less than .05 indicating significant differences.

## Results

### Participant Characteristics Between the Virological Relapse and non-Virological Relapse Groups

A total of 112 patients with CHB were initially HBeAg positive and started to receive ETV treatment. Drug discontinuance was allowed for 112 CHB patients who were HBeAg-positive before treatment and received ETV treatment after 12-month consolidation period during which patients achieved HBV DNA negative conversion and HBeAg seroconversion. Among them, 6 patients returned to the local hospital and thus were unable to come to the hospital regularly, 7 patients refused to follow up, and 1 patient had an unintended pregnancy. A total of age- and sex-matched 100 healthy controls were included in this study. The off-treatment VR rates at the 6th month, 9th month, and 12th month were 33.7% (33/98), 10.2% (10/98), and 9.2% (9/98), respectively. The cumulative VR rate was 53.1% (52/98) at 12 months after discontinuance of ETV treatment. Thus, the VR group had 52 patients and the non-VR group had 46 patients. Patients’ characteristics between the VR and non-VR groups are presented in Table 1. Compared to the non-VR group, a greater HBV DNA load was only demonstrated in the VR group (*P* = .016).

### Serum miR-146a and miR-155 in the Virological Relapse and non-Virological Relapse Groups Before Starting Entecavir Treatment

**Figure 1.**
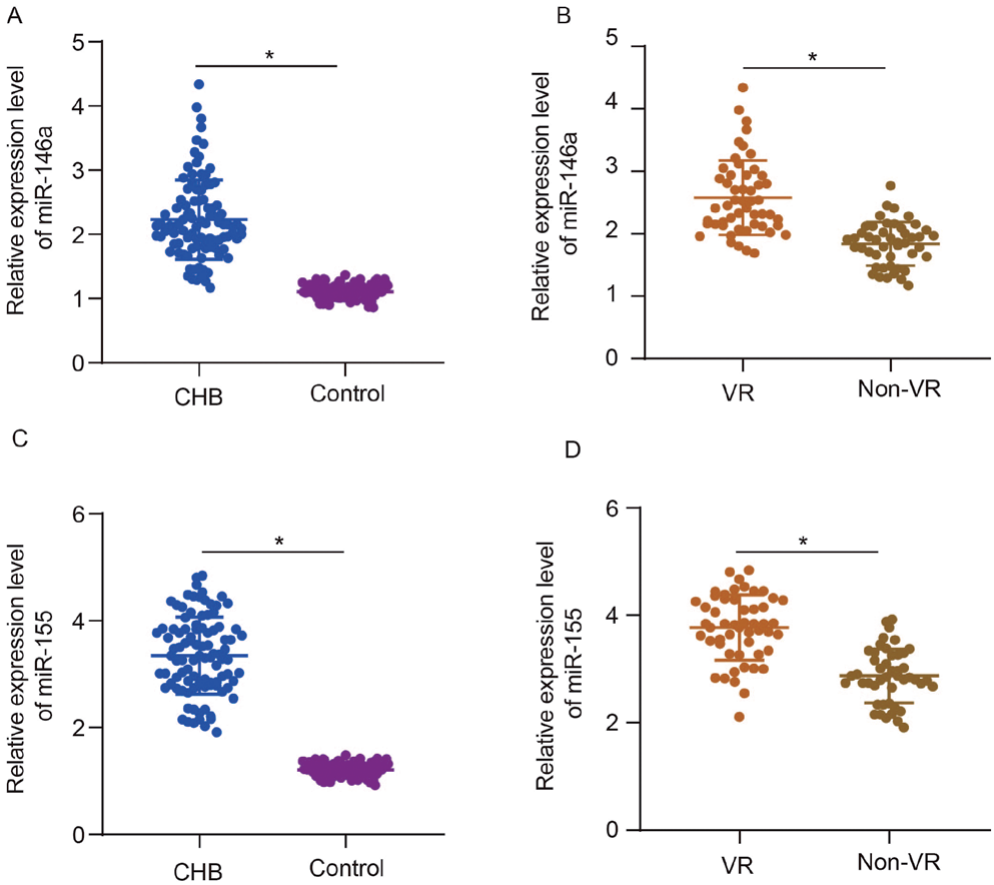
Relative expression levels of serum miR-146a and miR-155 between control and CHB groups, between the VR and non-VR groups before starting ETV treatment. A, miR-146a (CHB vs. control). B, miR-146a (VR vs. non-VR). C, miR-155 (CHB vs. control). D, miR-155 (VR vs. non-VR). *P < .05 analyzed by independent sample *t-*test, *VR, virologic response; M, month.

The relative expression level of miR-146a in the cohort of patients with CHB was 2.23 (mean value) and that of miR-146a in control was 1.11 (mean value) (Figure 1A). The relative expression level of miR-155 in the cohort of patients with CHB was 3.35 (mean value) and that of miR-155 in control was 1.21 (mean value) (Figure 1B). Compared to control, CHB patients were observed with increases in serum miR-146a and miR-155 before starting ETV treatment (*P* < .001). The relative expression level of miR-146a in the VR group was 2.58 (mean value) and that of miR-146a in the non-VR group was 1.84 (mean value) (Figure 1C). The relative expression level of miR-155 in the VR group was 3.77 (mean value) and that of miR-155 in the non-VR group was 2.87 (mean value) (Figure 1D). The VR group was demonstrated with increases in serum miR-146a and miR-155 level than the non-VR group before starting ETV treatment (*P* < .001).

### Serum miR-146a and miR-155 in the Virological Relapse and Non-Virological Relapse Groups After Entecavir Discontinuance

**Figure 2.**
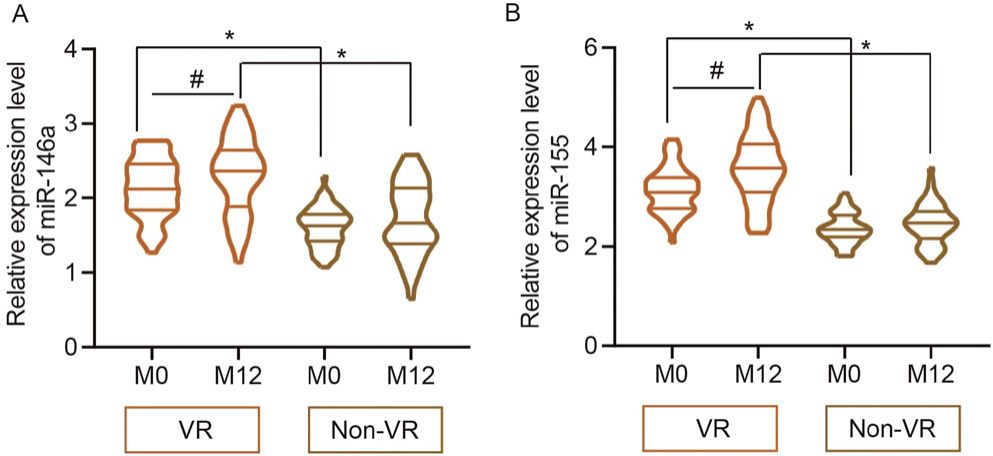
Relative expression levels of serum miR-146a and miR-155 between the VR and non-VR groups after ETV discontinuance. A, [VR (M0 vs. M12) vs. non-VR (M0 vs. M12)]. B, miR-155 [VR (M0 vs. M12) vs. non-VR (M0 vs. M12)]. M0 indicates the beginning of ETV discontinuance and M12 indicates 12 months after ETV discontinuance. *P < .05 analyzed by independent sample *t*-test and #*P* < .05 analyzed by dependent sample *t*-test, *VR, virologic response; M, month.

The relative expression level of miR-146a at the beginning of ETV discontinuance in the VR group was 2.13 (mean value) and that of miR-146 in the non-VR group was 1.58 (mean value). The relative expression level of miR-146a at 12 months after ETV discontinuance in the VR group was 2.30 and that of miR-146 in the non-VR group was 1.62. The VR group showed an increase in serum miR-146a at 12 months after ETV discontinuance (*P* = .008) but the non-VR group not (*P* = .342). Whether at the beginning of ETV discontinuance or at 12 months after ETV discontinuance, the VR group was demonstrated with an elevation in serum miR-146a compared to the non-VR group (*P* < .001, Figure 2A).

The relative expression level of miR-155 at the beginning of ETV discontinuance in the VR group was 3.14 (mean value), while that of miR-155 in the non-VR group was 2.37 (mean value). The relative expression level of miR-155 at 12 months after ETV discontinuance in the VR group was 3.57 (mean value), while that of miR-155 in the non-VR group was 2.47 (mean value). The VR group showed an increase in serum miR-146a at 12 months after ETV discontinuance (*P* < .001), but the non-VR group not (*P* = .258). Whether at the beginning of ETV discontinuance or at 12 months after ETV discontinuance, the VR group was demonstrated with an elevation in serum miR-155 compared to the non-VR group (*P* < .001, Figure 2B).

### Serum miR-146a and miR-155 Levels as Predictive Tools for Virological Relapse of Chronic Hepatitis B Patients After Entecavir Discontinuance

**Figure 3.**
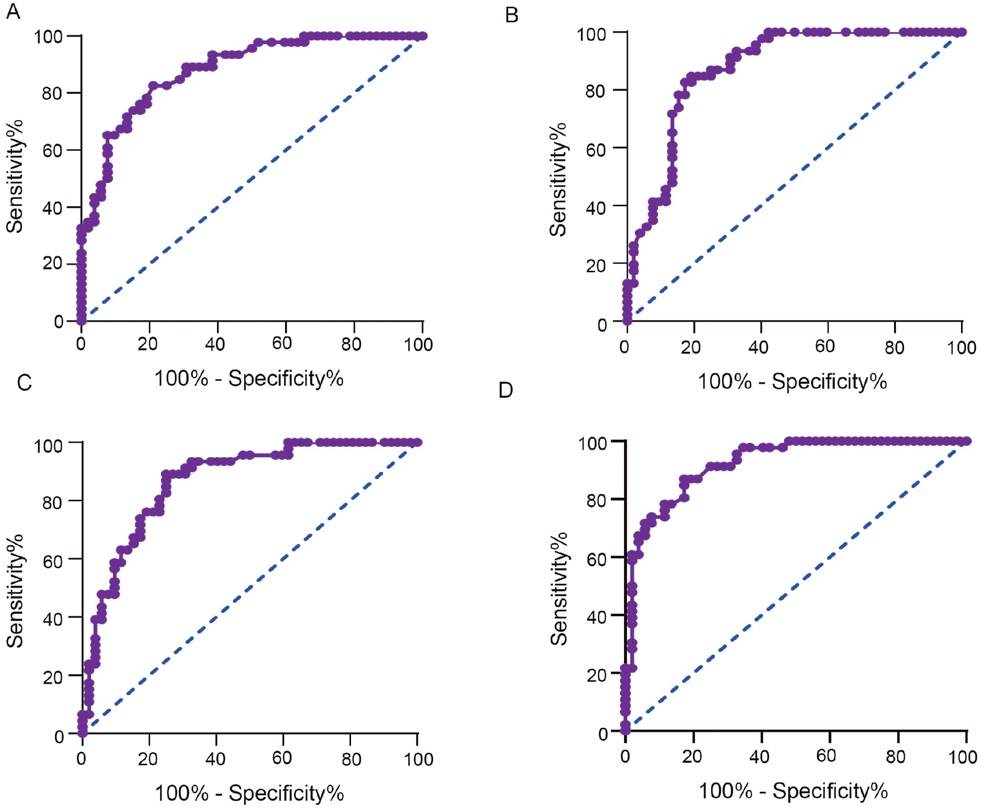
Serum miR-146a and miR-155 levels before starting ETV treatment and at the beginning of ETV discontinuance as predictive tools for VR after ETV discontinuance. A, miR-146a before starting ETV treatment. B, miR-146a at the beginning of ETV discontinuance. C, miR-155 before starting ETV treatment. D, miR-155 at the beginning of ETV discontinuance.

The ROC curve of serum miR-146a before starting ETV treatment in a predictive test for VR after ETV discontinuance is plotted in Figure 3A (AUC: 0.881; sensitivity: 78.8%; specificity: 82.6%). The ROC curve of serum miR-146a at the end of ETV treatment in a predictive test for VR after ETV discontinuance is plotted in Figure 3B (AUC: 0.875; sensitivity: 80.8%; specificity: 84.8%). The ROC curve of serum miR-155 before starting ETV treatment in a predictive test for VR after ETV discontinuance is plotted in Figure 3C (AUC: 0.866; sensitivity: 75.0%; specificity: 89.1%). The ROC curve of serum miR-155 at the end of ETV treatment in a predictive test for VR after ETV discontinuance is plotted in Figure 3D (AUC: 0.922; sensitivity: 82.7%; specificity: 87.0%). Table 2 lists relevant data of serum miR-146a and miR-155 levels as predictive tools for VR after ETV discontinuance.

## Discussion

Sustained HBsAg loss is rarely achieved with the present NA treatments, which creates a critical need to identify accurate predictors to reflect the effect of NA therapy and course of NA, thus optimizing treatment using the roadmap model.^15^ Increasing numbers of studies have reported that miRNAs both in serum and HBsAg-particles are associated with HBV replication and activation, and that HBV could alter expressions of miRNAs, including miR-146a and miR-155.^16^^,17^ This finding led to an intriguing hypothesis that miR-146a and miR-155 could be used to early evaluate the efficacy of antiviral therapy to control viral loads and identify sustained-virological-responders after stopping ETV treatment in CHB patients. To prove this hypothesis, the study focused on circulating miR-146a and miR-155 for their potential as predictors of virological response in CHB patients after ETV discontinuance. Our study demonstrated increased relative expression levels of serum miR-146a and miR-155 in HBeAg-positive CHB patients with VR after 12-month consolidation therapy compared to those without VR for 12 months following ETV discontinuance.

The most recently revised APASL guidelines (2015) recommend that HBeAg-positive CHB patients who have achieved HBeAg seroconversion with undetectable serum HBV DNA receive consolidation therapy for an additional 12 months or more.^14^ In Dai et al’s^18^ study, HBeAg-positive CHB patients showed response rates (HBeAg seroconversion with undetectable serum HBV DNA) of 25.6%, 39.0%, and 71.4%, respectively, at 6-month follow-up after consolidation durations of 12 months, 12-18 months, and 18 months. Their results showed 12-month consolidation therapy or more allowed HBeAg-positive patients to achieve HBeAg seroconversion with undetectable serum HBV DNA. Huang et al^19^ have shown that the durable virological remission of HBeAg-positive CHB patients at 52 weeks after ETV discontinuance was 51.8% (58/112). Sun et al^20^ reported that the sustained virological response rate of 3-year consolidation therapy was 36.59% (30/82) after 1-year follow-up. In our study, HBeAg-positive CHB patients were withdrawn from ETV treatment after 12-month consolidation therapy, and the maintenance of the response was 46.9% (46/98) at 12 months after discontinuance of ETV treatment, which was partially consistent with other studies.

Earlier work demonstrated a serum miRNA signature that is related to both naturally occurring and therapy-induced immune control of CHB, supporting that miRNAs expressed in the circulation might be a useful screening approach for patients who could achieve a sustained switch from CHB to inactive HBV infection after antiviral treatment.^21^ Previous evidence showed miR-146a was upregulated in HBV-expressing HepG2.2.15 cells compared to HepG2 cells, suggesting its association with HBV replication and expression.^22^ Not only in cell experiments, but Liu et al^23^ found miR-146a could maintain immune tolerance and facilitate HBV persistence in an HBV carrier mouse model. High levels of miR-146a may be important for maintaining HBV replication and reactivation after antiviral therapy by eradicating the immune control, thus leading to the aggravation of CHB.^24^ As our results of miR-146a detection demonstrated, miR-146a levels were significantly increased in the VR group compared to the non-VR group before initiating ETV treatment and after 12-month consolidation therapy, indicating persistent elevations in serum miR-146a levels may be associated with viral relapse in CHB patients after discontinuance of ETV treatment. The evidence of miR-155 reinforcing HBV replication has been proved in other study.^25^ Hepatitis B virus-positive tumors, livers obtained from HBV transgenic mice, and HBV X gene-overexpressing hepatoma cell lines were all found to highly express miR-155 levels, indicating the involvement of miR-155 in the development of CHB and HBV-related HCC.^17^^,^^26^^,27^ Zhang et al^28^ evaluated the effect of pegylated interferon-α, a therapeutic approach for CHB, on miR-155 expression in hepatoma cells and found that pegylated interferon-α could efficiently inhibit the expression of miR-155 to prevent HCC progression. miR-155 was experimentally studied as a target of resveratrol to inhibit HBV replication in animal models of HBV infection, which indirectly showed the targeted inhibition of miR-155 in relation to virological response after antiviral therapy.^29^ In our study, we detected higher miR-155 levels in the VR group than the non-VR group before initiating ETV treatment and after 12-month consolidation therapy, suggesting persistent elevations in serum miR-155 levels may be associated with viral relapse in CHB patients after discontinuance of ETV treatment.

Several limitations should be discussed. One of the limitations is the absence of a longer consolidation period, such as 18 months, and thus the effects of consolidation therapy duration (12 months vs. 18 months) on miR-146a and miR-155 expressions are not discussed. Another limitation is no measurement of miR-146a and miR-155 in liver specimens, as only a few patients accepted liver biopsies. Lastly, the relatively small patient sample size should be considered when the data were interpreted. Any new findings, such as the difference in miR-146a and miR-155 expressions between 12 months and 18 months of consolidation therapy, or between these expressions in the serum samples and liver tissues, need to be confirmed in further studies with a large-scale population.

In conclusion, our study provides evidence suggesting that elevations of miR-146a and miR-155 levels in the circulation may be linked to viral relapse in CHB patients who are positive for HBeAg after ETV discontinuance. The study uncovers potential candidate miRNAs for the early identification of patients with a sustained switch from CHB to inactive HBV infection after NA therapy. We suggest emphasizing the need for close monitoring of CHB patients with elevations of miR-146a and miR-155 in circulation. Future studies could explore whether long-term consolidation therapy or combination antiviral strategies would be beneficial for this patient population.

## Figures and Tables

**Table 1.** Characteristics of Patients Between VR and Non-VR Groups at Baseline

Characteristic	Total (n = 98)	VR (n = 52)	Non-VR (n = 46)	*P*
Age (year, mean [SD])	34.4 (9.2)	35.4 (9.3)	33.2 (9.1)	.230
Sex (male, n [%])	61 (62.2%)	33 (63.5%)	28 (60.9%)	.837
Family history of liver cancer (n [%])	8 (8.2%)	6 (11.5%)	2 (4.3%)	.276
Course of treatment (month, mean [SD])	30.8 (3.2)	30.4 (3.1)	31.4 (2.9)	.109
ALT (IU/L), (<100/100-199/200≥, n)	36/23/39	15/15/22	21/8/17	.181
HBV-DNA (Log10 copy/mL), (<6/6-7.6/7.6>, n)	8/35/55	2/14/35	6/21/19	.016
HBsAg ([log10 IU/mL], mean [SD])	3.77 (0.71)	3.88 (0.82)	3.64 (0.53)	.100

Quantitative variables were assessed using the unpaired *t*-test and count data were assessed using the chi-squared tests.

ALT, alanine aminotransferase; VR, Virologic relapse.

**Table 2.** Serum miR-146a and miR-155 Levels as Predictive Tools for VR After ETV Discontinuance

Variables	Area	*P*	95% CI	
			Lower Bound	Upper Bound
Relative expression level of miR-146a before starting ETV treatment	0.881	<.001	0.816	0.946
Relative expression level of miR-146a at the beginning of ETV discontinuance	0.875	<.001	0.806	0.945
Relative expression level of miR-155 before starting ETV treatment	0.866	<.001	0.795	0.938
Relative expression level of miR-155 at the beginning of ETV discontinuance	0.922	<.001	0.871	0.973

## Data Availability

The data supporting the findings of this study are available within the article.
